# Fístula Átrio-Esofágica após Ablação por Cateter de Fibrilação Atrial: Podemos Realmente Preveni-la?

**DOI:** 10.36660/abc.20250148

**Published:** 2025-05-14

**Authors:** Luiz Eduardo Montenegro Camanho

**Affiliations:** 1 Hospital Pró-Cardíaco Rio de Janeiro RJ Brasil Hospital Pró-Cardíaco - Serviço de Arritmias e Estimulação Cardíaca Artificial, Rio de Janeiro, RJ - Brasil

**Keywords:** Fístula Esofágica, Fibrilação Atrial, Ablação por Cateter

A ablação por cateter de fibrilação atrial (FA) é um procedimento amplamente utilizado e estabelecido na prática clínica. Diversos estudos randomizados já demonstraram a superioridade deste método em pacientes com FA paroxística/ persistente, sintomáticos e refratários a terapia antiarrítmica, com forte impacto positivo na qualidade de vida e na redução da recorrência clínica desta arritmia, independentemente do tipo de energia utilizada.^1 – 3^ O objetivo primário do procedimento é o isolamento elétrico das veias pulmonares, associada ou não a lesões mais extensas, em particular, da parede posterior.

As principais complicações relacionadas ao procedimento, tais como, morte, evento tromboembólico per-procedimento, fístula átrio-esofágica (FAE), tamponamento cardíaco, estenose grave de veia pulmonar e paralisia permanente de nervo frênico são, na realidade, complicações infrequentes (0,02 a 1,3%). Uma recente publicação demonstrou um decréscimo destas complicações nos últimos anos (2018-2022) quando comparado aos cinco anos antecedentes (3,8 versus 5,3%).^4^

A FAE é uma complicação rara e potencialmente letal após ablação por cateter de FA, tendo sido observada desde os primeiros anos após introdução do procedimento na prática clínica.^5^ As manifestação clínicas relacionadas são febre, dor torácica, odinofagia, sintomas neurológicos por embolia séptica, hematêmese, choque séptico e derrame pericárdico purulento (fístula esôfago-pericárdica).^6^ A incidência descrita varia de 0,02 a 0,1%.^7^ O estudo POTTER-AF, o maior registro multicêntrico sobre FAE após ablação de FA, reuniu 553.279 procedimentos de ablação (radiofrequência: 62,9%; crioablação: 36,2% e outras modalidades: 0,9%) em 214 centros de 35 países. Foram registrados 138 casos de FAE (0,025%) e, a ocorrência deste evento foi significativamente maior no grupo da radiofrequência em relação a crioablação (0,038 x 0,0015%, p < 0,0001). O sintoma inicial mais comum foi febre (59,3%) e o diagnóstico foi estabelecido pela tomografia computadorizada de tórax em 80,2% dos pacientes. O tempo médio de aparecimento dos sintomas foi de 18 dias e para o diagnóstico foram de 21 dias após o procedimento. A mortalidade descrita foi elevada e de 65,8%. A análise multivariada demonstrou que os fatores associados a sobrevida nesta população foram a utilização de monitorização esofágica (p=0,012), tratamento cirúrgico da FAE (p=0,027) e o tipo de anestesia (sedação consciente; p=0,030).^8^

Inúmeras estratégias já foram propostas com o objetivo de reduzir o risco de FAE após ablação de FA: visualização do posicionamento e relação do esôfago com a parede posterior do átrio esquerdo através dos sistemas de mapeamento eletro-anatômico ou ecocardiografia intra-cardíaca; evitar lesões consecutivas na parede posterior em regiões adjacentes ao esôfago; resfriamento esofágico; desvio mecânico do esôfago; monitorização contínua da temperatura esofágica; utilização do ecocardiograma intra-cardíaco; redução da força de contato na parede posterior e prescrição de inibidores de bomba de prótons. A última, apesar de ser muito frequentemente utilizada, ainda carece de evidências que comprovem o seu real benefício.^9^

Sanchez et al.^10^ descreveram uma série multicêntrica que envolveu 25 centros e 14.224 pacientes em que foi adotado o resfriamento esofágico durante a ablação de FA. Apesar da incidência de apenas 0,146% da ocorrência de FAE no grupo pré resfriamento do esôfago, esta estratégia levou a redução significativa (p<0,0001) deste tipo de complicação.

O estudo OPERA randomizou prospectivamente 200 pacientes submetidos a ablação de FA e endoscopia digestiva alta pós-procedimento, em dois grupos: 100 pacientes - ablação por radiofrequência com monitorização esofágica vs. 100 pacientes: não foi utilizado monitorização esofágica e definida uma potência fixa de 25W para aplicação na parede posterior. Os resultados deste ensaio randomizado demonstraram que a monitorização da temperatura esofágica não afetou a probabilidade de lesões esofágicas detectadas endoscopicamente, assim como o pico de temperatura observado. A redução empírica da potência na parede posterior não influenciou a eficácia do procedimento.^11^

Desta forma, se pode observar que a questão da prevenção efetiva de FAE é um assunto absolutamente em aberto na literatura médica e principalmente relacionada a ocorrência muito baixa deste tipo de complicação, o que dificulta a condução de grandes ensaios clínicos pelo número insuficiente de amostras negativas.

Nesta revista, Ferraz et al.^12^ apresentaram um estudo unicêntrico retrospectivo que envolveu 823 pacientes submetidos a avaliação endoscópica sistemática após ablação de FA em procedimentos consecutivos realizados entre 2016 e 2022. O objetivo foi apresentar a experiência de 7 anos de um monitoramento endoscópico sistemático de lesão esofágica após ablação por cateter de FA. A maioria (n=588; 71,4%) dos pacientes submetidos ao procedimento era do sexo masculino, e 575 (69,9%) na forma de FA paroxística. O monitoramento da temperatura esofágica foi realizado usando um sensor único em 310 pacientes (40,3%) e uma sonda multissensor em 306 (39,8%) pacientes. A esofagogastroduodenoscopia (EGD) foi realizada em até 7 dias após a ablação, sendo a maioria no dia seguinte ao procedimento. As lesões estavam presentes em 217 EGD (26,5%): hematoma-equimose em 27 (3,3%), eritema em 14 (1,7%), erosão em 78 (9,5%) e úlcera em 67 (8,2%) dos pacientes. Nenhuma estratégia de proteção do esôfago foi associada à maior ocorrência de úlceras, com exceção do uso de cateter de ponta de 8mm (14,7% de úlceras com cateter de ponta de 8mm vs. 6,7% com outros cateteres, p = 0,001). Lesões térmicas foram detectadas precocemente e tratadas. A maioria das lesões foi considerada curada na endoscopia, mas um paciente que foi submetido ao isolamento da veia pulmonar com um cateter de ponta de 8mm apresentou fístula esofágica, que foi tratada com sucesso com clipe metálico endoscópico e técnica *endoloop* .

Os autores concluem então que a incidência de lesões esofágicas é alta na EGD de rotina realizada após a ablação de FA, embora, na maioria dos casos, sua cura ocorra espontaneamente. Pacientes que se submeteram à ablação com o cateter de ponta de 8mm apresentaram lesões térmicas mais graves. Endoscopia esofágica precoce pode ajudar no diagnóstico de lesões em fases iniciais e na prevenção de fístulas após a ablação de FA.

Baseado nos dados apresentados pelos autores, a EGD traz informações importantes e inquestionáveis quanto a ocorrência de lesões esofágicas, porém a relevância clínica destes achados e a aplicabilidade do método de forma rotineira fica difícil de ser estabelecida, já que na amostra de 823 pacientes, apenas 1 paciente evoluiu com FAE (0,1%). Na verdade, a muito baixa ocorrência deste tipo de evento é felizmente o verdadeiro motivo de até hoje não termos definido a estratégia ideal para a prevenção de uma complicação tão temida. Vale ressaltar também que os cateteres de ponta 8 mm, embora ainda utilizados, vem sendo progressivamente e rapidamente descontinuados e substituídos pelos cateteres irrigados e com sensores de força de contato.

Enquanto esta questão não se resolve por completo, fica a expectativa quanto aos resultados da ablação por campo pulsado, em que, teoricamente, haveria uma maior seletividade do tecido miocárdico e sem lesões a órgãos adjacentes.^13^ Caso se confirme esta hipótese, esta forma de energia trará sem dúvida um substancial avanço no tratamento percutâneo dos pacientes portadores de FA.
