# Supplementary material for: Fístula Átrio-Esofágica após Ablação por Cateter de Fibrilação Atrial: Podemos Realmente Preveni-la?
Source: Arq Bras Cardiol. 2025 May 14;122(3):e20250148. [Article in Portuguese] doi: 10.36660/abc.20250148 (PMC12101577; doi:10.36660/abc.20250148)
Supplement: Translation

# Translation: Atrioesophageal Fistula After Catheter Ablation of Atrial Fibrillation: Can We Really Prevent It?

**DOI:** 10.36660/abc.20250148i

**Authors:** Luiz Eduardo Montenegro Camanho

**Affiliations:** 1 Rio de Janeiro RJ Brazil Hospital Pró-Cardíaco - Serviço de Arritmias e Estimulação Cardíaca Artificial, Rio de Janeiro, RJ - Brazil

**Keywords:** Esophageal Fistula, Atrial Fibrillation, Catheter Ablation

Catheter ablation of atrial fibrillation (AF) is a widely used and established procedure in clinical practice. Several randomized studies have demonstrated the superiority of this method in patients with paroxysmal/persistent AF who are symptomatic and refractory to antiarrhythmic therapy, with a strong positive impact on quality of life and in reducing the clinical recurrence of this arrhythmia, regardless of the type of energy used.^1 – 3^The primary objective of the procedure is the electrical isolation of the pulmonary veins, associated or not with more extensive lesions, particularly of the posterior wall.

The main complications related to the procedure, such as death, per-procedure thromboembolic event, atrioesophageal fistula (AEF), cardiac tamponade, severe pulmonary vein stenosis, and permanent phrenic nerve palsy are, in reality, infrequent complications (0.02 to 1.3%). A recent publication demonstrated a decrease in these complications in recent years (2018-2022) when compared to the previous five years (3.8 versus 5.3%).^4^

AF is a rare and potentially lethal complication after catheter ablation of atrial fibrillation, having been observed since the first years after the introduction of the procedure into clinical practice.^5^The related clinical manifestations are fever, chest pain, odynophagia, neurological symptoms due to septic embolism, hematemesis, septic shock, and purulent pericardial effusion (esophageal-pericardial fistula).^6^The incidence reported ranges from 0.02 to 0.1%.^7^The POTTER-AF study, the largest multicenter registry of AF after AF ablation, included 553,279 ablation procedures (radiofrequency: 62.9%; cryoablation: 36.2% and other modalities: 0.9%) in 214 centers in 35 countries. A total of 138 cases of AEF (0.025%) were recorded, and the occurrence of this event was significantly higher in the radiofrequency group compared to cryoablation (0.038 x 0.0015%, p < 0.0001). The most common initial symptom was fever (59.3%), and the diagnosis was established by chest computed tomography in 80.2% of patients. The mean time for the onset of symptoms was 18 days, and for diagnosis was 21 days after the procedure. The mortality rate was high, at 65.8%. Multivariate analysis demonstrated that the factors associated with survival in this population were the use of esophageal monitoring (p = 0.012), surgical treatment of AEF (p = 0.027), and the type of anesthesia (conscious sedation; p = 0.030).^8^

Numerous strategies have been proposed to reduce the risk of AEF after AF ablation: visualization of the positioning and relationship of the esophagus with the posterior wall of the left atrium through electroanatomical mapping systems or intracardiac echocardiography; avoidance of consecutive lesions in the posterior wall in regions adjacent to the esophagus; esophageal cooling; mechanical esophageal bypass; continuous monitoring of esophageal temperature; use of intracardiac echocardiography; reduction of contact force on the posterior wall; and prescription of proton pump inhibitors. The latter, despite being very frequently used, still lacks evidence to prove its real benefit.^9^

Sanchez et al.^10^described a multicenter series involving 25 centers and 14,224 patients in which esophageal cooling was adopted during AF ablation. Despite the incidence of only 0.146% of AEF in the pre-esophageal cooling group, this strategy led to a significant reduction (p<0.0001) of this type of complication^10^.

The OPERA study prospectively randomized 200 patients undergoing AF ablation and post-procedure upper gastrointestinal endoscopy into two groups: 100 patients - radiofrequency ablation with esophageal monitoring vs. 100 patients: no esophageal monitoring was used, and a fixed power of 25 W was defined for application to the posterior wall. The results of this randomized trial demonstrated that esophageal temperature monitoring did not affect the probability of endoscopically detected esophageal lesions, as well as the observed peak temperature. Empirical reduction of power in the posterior wall did not influence the efficacy of the procedure.^11^

Thus, it can be observed that the issue of effective prevention of AED is an absolutely open subject in the medical literature and is mainly related to the very low occurrence of this type of complication, which makes it difficult to conduct large clinical trials due to the insufficient number of negative samples.

In this journal, Ferraz et al.^12^presented a retrospective single-center study involving 823 patients who underwent systematic endoscopic evaluation after AF ablation in consecutive procedures performed between 2016 and 2022. The objective was to present the 7-year experience of systematic endoscopic monitoring of esophageal injury after catheter ablation of AF. The majority (n=588; 71.4%) of patients undergoing the procedure were male, and 575 (69.9%) had paroxysmal AF. Esophageal temperature monitoring was performed using a single sensor in 310 patients (40.3%) and a multisensor probe in 306 (39.8%) patients. Esophagogastroduodenoscopy (EGD) was performed within 7 days after ablation, with the majority performed on the day after the procedure. Lesions were present in 217 EGD (26.5%): hematoma-ecchymosis in 27 (3.3%), erythema in 14 (1.7%), erosion in 78 (9.5%) and ulcer in 67 (8.2%) of patients. No esophageal protection strategy was associated with a higher incidence of ulcers, with the exception of the use of an 8-mm tip catheter (14.7% ulcers with an 8-mm tip catheter vs. 6.7% with other catheters, p = 0.001). Thermal injuries were detected early and treated. Most injuries were considered healed at endoscopy, but one patient who underwent pulmonary vein isolation with an 8-mm tip catheter developed an esophageal fistula, which was successfully treated with an endoscopic metal clip and endoloop technique.

The authors conclude that the incidence of esophageal injuries is high in routine EGD performed after AF ablation, although in most cases, their healing occurs spontaneously. Patients who underwent ablation with the 8-mm tip catheter presented more severe thermal injuries. Early esophageal endoscopy may help diagnose lesions at early stages and prevent fistulas after AF ablation.

Based on the data presented by the authors, EGD provides important and unquestionable information regarding the occurrence of esophageal lesions. However, the clinical relevance of these findings and the routine applicability of the method are difficult to establish since in the sample of 823 patients, only 1 patient developed AEF (0.1%). In fact, the very low occurrence of this type of event is, fortunately, the real reason why, to date, we have not defined the ideal strategy for preventing such a feared complication. It is also worth mentioning that 8 mm tip catheters, although still used, have been progressively and rapidly discontinued and replaced by irrigated catheters with contact force sensors.

Until this issue is completely resolved, expectations remain regarding the results of pulsed-field ablation, in which, theoretically, there would be greater selectivity of myocardial tissue and no damage to adjacent organs.^13^If this hypothesis is confirmed, this form of energy will undoubtedly bring a substantial advance in the percutaneous treatment of patients with AF.
